# Bactericidal Effect of Synthetic Phenylalkylamides Inspired by Gibbilimbol B Against *Neisseria gonorrhoeae*

**DOI:** 10.3390/molecules30112406

**Published:** 2025-05-30

**Authors:** Larissa V. F. Oliveira, Andre G. Tempone, Myron Christodoulides, Joao Henrique G. Lago

**Affiliations:** 1 Center of Natural Sciences and Humanities, Federal University of ABC, Sao Paulo 09210-580, Brazil; larissavfoliveira@hotmail.com; 2 Neisseria Research Laboratory, Molecular Microbiology, School of Clinical and Experimental Sciences, Faculty of Medicine, University of Southampton, Southampton SO16 6YD, UK; 3 Laboratory of Pathophysiology, Butantan Institute, Sao Paulo 05508-040, Brazil; andre.tempone@butantan.gov.br

**Keywords:** gibbilimbol B, natural products, analogs, *Neisseria gonorrhoeae*, bactericidal

## Abstract

Increasing multidrug resistance in *Neisseria gonorrhoeae* poses a serious and escalating public health crisis. The World Health Organization has classified *N. gonorrhoeae* as a high-priority pathogen for developing new antimicrobials. Natural products provide a promising avenue for antimicrobial discovery, serving as direct therapeutic agents or prototypes for novel drug development. Among these, gibbilimbol B, a compound isolated from *Piper malacophyllum*, is particularly attractive due to its biological potential and simple structure. In this study, eight synthetic phenylalkylamides (**1**–**8**) inspired by gibbilimbol B were synthesized and evaluated for their antibacterial activity against *N. gonorrhoeae*. The in vitro bacterial assays revealed that these compounds exhibit notable antibacterial activity, including against resistant strains selected from the CDC/FDA antimicrobial panel (strains AR-173, AR-174, AR-187, and AR-200). All synthesized compounds demonstrated superior efficacy in killing *N. gonorrhoeae* compared to gibbilimbol B. Notably, compound **8** [(*E*)-4-chloro-*N*-(oct-4-en-1-yl)benzamide] showed an MBC50 of 6.25 µM, representing a four-fold improvement in bactericidal activity over the natural compound. This study represents the first exploration of gibbilimbol analogs for antibacterial applications, highlighting the novelty of the work and paving the way for the development of new antibacterial agents.

## 1. Introduction

Antimicrobial resistance (AMR) represents a serious global public health problem, currently associated with nearly 700,000 deaths annually. Without further intervention, projections indicate that this number could rise to 10 million per annum by 2050 [1,2]. The widespread use and misuse of antibiotics and the natural adaptability of microorganisms to antibiotics has facilitated the emergence of resistant strains. Consequently, the search for alternative treatment methods against these microorganisms is urgent. To address this urgent problem, the World Health Organization (WHO) published a list of antibiotic-resistant priority pathogens to guide and stimulate research and development in this field [3,4]. This list includes 15 bacterial families that pose the greatest risk to human health, for which the development of new antimicrobials is critically needed. *Neisseria gonorrhoeae*, a pathogen that presents distinct public health challenges, was included in the high-priority category within the list [5].

*Neisseria gonorrhoeae* is the causative agent of the sexually transmitted disease gonorrhoeae, with around 87 million cases reported annually worldwide, primarily from least-developed countries [6]. There are no vaccines to prevent gonococcal infections, and control has relied on the use of antibiotics. However, gonococci have developed resistance to all antibiotics that are currently available, and monotherapy with ceftriaxone is the current recommendation from the Centers for Disease Control and Prevention (CDC, USA) and the British Association for Sexual Health and HIV (BASHH) for treating gonorrhea. However, the increase in resistance to ceftriaxone now poses a serious threat [7]. Therefore, the search for alternative treatments against gonococcus is necessary and urgent, and one option is to develop new antimicrobials based on natural products [8].

Natural products have shown great potential as antimicrobials, even against some multidrug-resistant strains, and they have already been studied as alternatives to current drugs [9,10]. Indeed, out of the 162 antibacterial agents approved by the Food and Drug Administration (FDA), USA, from 1981 to 2019, about 50% originate from natural products and their derivatives, which emphasizes their relevance to drug discovery [11]. Furthermore, in recent studies, various natural product extracts, essential oils, and isolated compounds from medicinal plants have been explored and have shown promising potential against *N. gonorrhoeae* [12,13,14,15]. Natural products can be used in natura or they can be used as prototypes for designing new molecules with improved pharmacological potential [16]. In this sense, the natural product gibbilimbol B, isolated from the shrub *Piper malacophyllum* [17], can be considered as a hypothetical model for the design of analogs due to its biological potential and simple structure. Gibbilimbol B has demonstrated relevant biological activity [17,18,19,20,21,22], including anti-*Leishmania*, anti-*Trypanosoma*, anticancer, and antibacterial effects. Gibbilimbols showed bactericidal activity against *Mycobacterium bovis BCG* [21], *Staphylococcus epidermidis*, and *Bacillus cereus* [22]. Recently, studies have focused on designing and preparing synthetic gibbilimbol analogs and evaluating them as leishmanicidal and trypanocidal agents [23,24,25,26]. The chemical modifications explored in these studies included the introduction of different functional groups to the phenolic ring side chain, the substitution of the phenolic hydroxyl group with various *p*-substituents, and changes to the alkyl chain, such as length variations and unsaturation. Many of these modifications enhanced biological potency, reduced toxicity, and improved drug-likeness properties compared to the natural product [23,24,25,26]. Building on these promising results, in the current study, we synthesized various amides inspired by gibbilimbol B and tested the hypothesis that they showed activity against *Neisseria gonorrhoeae*. This represents the first novel testing of gibbilimbol and its analogs against gonococci to expand the repertoire of potential antimicrobial therapies.

## 2. Results and Discussion

### 2.1. Chemistry

**Figure 1:**
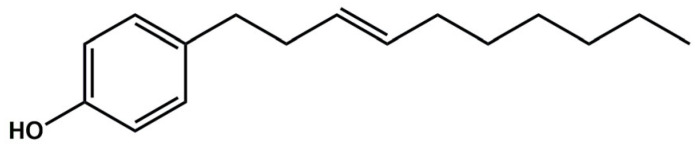
Chemical structure of gibbilimbol B.

Initially, eight amides chemically inspired by gibbilimbol B (Figure 1), a bioactive natural product obtained from *Piper malacophyllum*, were synthesized. The chemical variations between them included the presence or absence of a double bond at the C-4 position of the alkyl side chain, as well as modifications to the *p*-substituent on the aromatic ring. The *p*-substituents that were explored included electron-donating groups such as alkyl and methoxy, and electron-withdrawing groups such as fluoro and chloro. Thus, in this study, we assessed the bactericidal activity of amides **1**–**8** against *N. gonorrhoeae* and evaluated the influence of these chemical modifications on their bioactivity.

**Figure 2:**
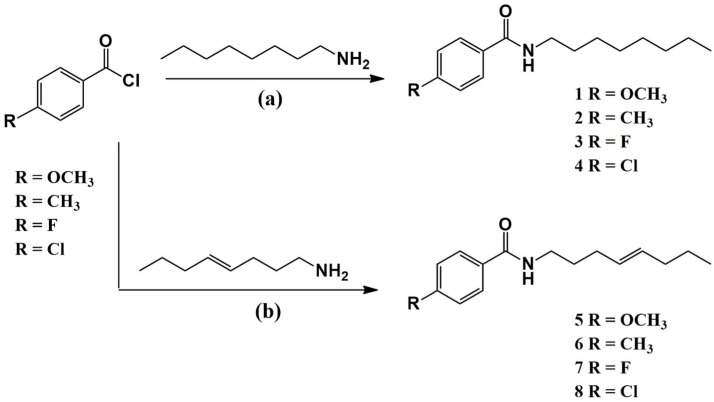
Illustration of the schematic procedure employed in the synthesis of the amides **1**–**8**. (**a**) Reaction with 1-octylamine; (**b**) reaction with (*E*)-oct-4-en-1-amine. Reaction conditions: CH_2_Cl_2_, 0 °C, N_2_ atmosphere, Et_3_N.

The synthesis of these compounds was based on a reaction between the different *p*-substituted benzoyl chlorides and the respective amine, 1-octylamine (a) or (*E*)-oct-4-en-1-amine (b) (Figure 2), following a procedure already described in the literature [25]. The compounds were obtained with moderate yield, and their structure was confirmed by nuclear magnetic resonance (NMR) spectroscopy and infrared spectroscopy (IR) (Supplementary Figures S1–S8).

### 2.2. Biological Evaluation

#### 2.2.1. Assessment of Anti-Gonococcal Activity

**Figure 3:**
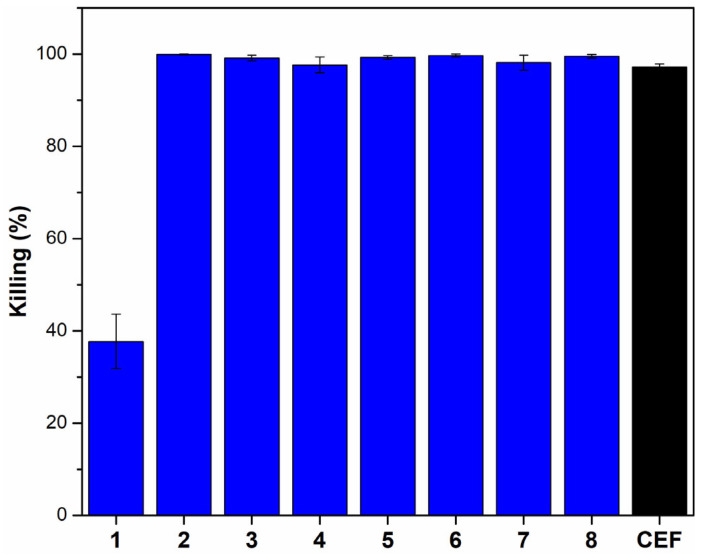
The bactericidal activity of gibbilimbol analogs **1**–**8** (blue) and ceftriaxone (CEF) (black) at 50 µM, assessed by viable counting in the bactericidal assay. The columns represent the mean % killing, and the error bars indicate the standard error of the mean (SEM) from four independent experiments (*n* = 4).

The antibacterial activity of compounds **1**–**8** was evaluated using an established in vitro minimum bactericidal activity (MBC) assay, through a viable counting method, against our laboratory reference *N. gonorrhoeae* strain, P9-17 [26,27]. Determinations of Minimum Inhibitory Concentration (MIC) values using standard broth microdilution assays could not be done, due to the instability of the compounds in the broth cultures, as demonstrated in pilot experiments. Ceftriaxone was used as a positive control. All compounds and antibiotics were initially tested at a screening concentration of 50 µM, and except for compound **1**, all of them killed >95% of gonococci, with values comparable to the positive control ceftriaxone (Figure 3). There were no significant differences in the bactericidal activities between all compounds **2**–**8** and ceftriaxone (*p* < 0.05), whereas the bactericidal activity of compound **1** was significantly lower than all the other compounds and the antibiotic (*p* > 0.05) (Figure 3).

**Figure 4:**
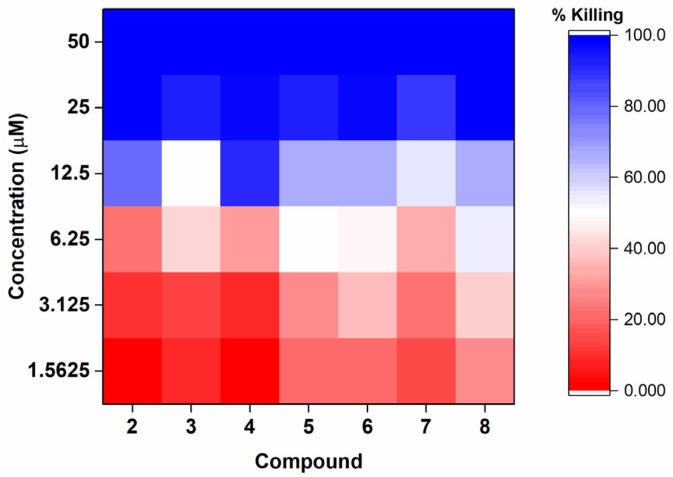
Heatmap showing the percentage of *N. gonorrhoeae* P9-17 killing at different concentrations (1.5625 to 50 µM) of gibbilimbol analogs **2**–**8**. Data represent the mean of three independent experiments, each performed in triplicate.

Next, the active compounds **2**–**8** were titrated against P9-17 to quantify their minimum bactericidal activity at 50% and 90% intervals, using a viable counting method as previously described (Figure 4) [27]. Titration experiments showed that the gibbilimbol analogs induced dose-dependent bactericidal effects and allowed the determination of MBC 50% and MBC 90% for each compound (Figure S9 and Table 1).

The gibbilimbol analogs required similar concentrations to kill 50% and 90% of the bacteria, ranging from 6.25 to 12.5 µM and 12.5 to 25.0 µM, respectively, depending on the compound. These values were comparable to those for the control ceftriaxone. All synthesized amides (except for the inactive compound **1**) demonstrated superior efficacy in killing gonococci compared to the natural product gibbilimbol B, with up to a four-fold increase in potency. These results suggest that the introduction of the amide group on the aromatic ring side chain potentially contributed to enhancing antibacterial activity.

Considering the related compounds **1** to **4**, which are amides with saturated alkyl side chains, and compounds **5** to **8**, which have unsaturated alkyl side chains (Figure 2), it is possible to evaluate the influence of different *p*-substituents on the aromatic ring. In general, compounds **2** to **4** exhibited similar antibacterial activity, suggesting that the nature of the substituents had limited impact within this series. Compound **1**, however, deviated from this pattern and was inactive under the tested conditions. A similar trend was observed among compounds **5** to **8**, which also showed comparable activity despite bearing different *para*-substituents. These results suggest that the substituent group on the aromatic ring was not a major determinant of activity in either series. Compound **1** did not reach a MBC50 value when tested at the screening dose of 50 µM, which suggests that reaching the MBC50 and MBC > 90 requires much higher concentrations, which would then exclude the original compound from further biological assessments. The reason for the lower activity may involve additional structural or physicochemical factors not directly assessed in this study, requiring further investigation, such as mechanistic or molecular interaction studies.

Furthermore, the impact of the presence or absence of unsaturation at the C-4 position of the alkyl side chain can be assessed by comparing compounds that share the same *p*-substituent group on the aromatic ring, but differ in this feature (**1**–**5**, **2**–**6**, **3**–**7**, and **4**–**8**). Compounds with a double bond at the C-4 position, which is similar in structure to the natural product gibbilimbol, exhibited marginal increases in bactericidal activity, although this difference was subtle across the range of MBC data generated within the replicate experiments, with only a two-fold increase at best. These results suggested that the presence of unsaturation in the alkyl side chain could potentially play a relevant role in antimicrobial activity that could be further explored in future studies for the development of new analogs. This finding aligns with previous reports in the literature, which have shown that unsaturation in the alkyl chain of various compounds, such as certain fatty acids, can enhance antibacterial activity [28,29,30,31,32].

Examining the bactericidal mechanism of gibbilimbol and its analogs was beyond the scope of this study; however, a plausible mode of action can be proposed. The natural product gibbilimbol B may possibly target bacterial cell membranes, disrupting their integrity and increasing permeability. This effect may be attributed to its amphipathic nature, with the hydroxyl group acting as a ‘polar head’ and the alkyl chain as a ‘lipophilic tail’ [17]. As observed for other amphiphilic compounds [33,34], this structural feature may enable gibbilimbol B to interact with both the lipid bilayer and the surrounding aqueous environment, facilitating membrane insertion and destabilization. This disruption can lead to the leakage of intracellular components and, ultimately, bacterial cell death. Regarding the gibbilimbol analogs, as previously mentioned, the amide analogs exhibited markedly higher bactericidal activity than the natural product, suggesting a distinct mode of action or the involvement of multiple pathways. The introduction of the amide group may have played a key role in enhancing antibacterial activity. Amides are known as effective molecular linkers, facilitating interactions with bacterial enzymes or membrane-associated proteins primarily through hydrogen bonding or electrostatic interactions [35,36]. Such interactions could interfere with essential bacterial processes, including cell wall synthesis or metabolic pathways, ultimately leading to bacterial death. A comprehensive investigation into the potential mechanism of action of these compounds is crucial for further development. Additional studies, such as membrane permeability assays, protein-binding analyses, and computational modeling, would be necessary to elucidate the precise mechanism underlying the activity of these compounds [37].

#### 2.2.2. Selected Compounds Tested Against Resistant *N. gonorrhoeae*

Resistance to the drugs commonly used as first-line treatment for gonococcal infections is a widespread global concern. In this context, the gibbilimbol analogs with more potent activity (i.e., **5**, **6**, and **8**) against the *N. gonorrhoeae* P9-17 laboratory strain were selected and tested against some gonococcal isolates from the CDC/FDA AR (Centers for Disease Control and Prevention (CDC) and the U.S. Food and Drug Administration (FDA-Antimicrobial Resistance Isolate Bank), which are reported to show increased MIC values towards ceftriaxone and azithromycin (Table 2). The analogs were tested at 6.25 µM and 25 µM, which were the MBC50 and MBC90 for P9-17, to evaluate whether the compounds retained bactericidal activity against resistant isolates.

Compounds **5**, **6**, and **8** effectively killed the resistant *N. gonorrhoeae* strains at the tested concentrations. Notably, compound **8** exhibited the most consistent performance across all isolates. This is a noteworthy observation, as resistance to ceftriaxone and reports of antibiotic treatment failures are becoming more frequent across many countries [38].

However, a minor limitation of our study was that we did not calculate the MBC values for the compounds **5**, **6**, and **8** tested against the resistant strains and, as mentioned above, broth microdilution assays could not be done with these compounds possibly due to interference from the complex broth ingredients. Thus, our bactericidal activity data cannot enable comparison with studies of other anti-gonococcal compounds reported in the literature, if a global standard is used, e.g., the Clinical and Laboratory Standards Institute (CLSI)-accredited broth microdilution assay to determine MIC. However, a caveat is that such comparisons of different compounds should be made side-by-side rather than by retrospectively analyzing outputs from the literature, where there may be several inter-laboratory confounders such as operator issues and reagent provenance.

## 3. Materials and Methods

### 3.1. General Procedures

All chemicals, such as octylamine (99%), triethylamine (99.5%), 4-Methoxybenzoyl chloride (99%), *p*-Toluoyl chloride (98%), 4-Fluorobenzoyl chloride (98%), and 4-Chlorobenzoyl chloride (99%), were purchased from Sigma-Aldrich (St. Louis, MO, USA) and used without any further purification. Dichloromethane (Synth, Brazil) was dried with phosphorus pentoxide, distilled, and stored under 4 Å molecular sieves prior to use. Nuclear magnetic resonance spectra (NMR) were acquired using an Ultrashield 300 spectrometer from Bruker (Billerica, MA, USA). Chloroform-d from Sigma-Aldrich (St. Louis, MO, USA) was used as the deuterated solvent and reference. IR spectra were recorded on a Cary 630 from Agilent (Santa Clara, CA, USA), on an ATR accessory with a spectral resolution of 4.0 cm^−1^.

### 3.2. Isolation of Gibbilimbol B from Piper malacophyllum

Information concerning the source, identification, and voucher number of the botanical material has been reported previously [17]. Dried and powdered leaves of *P. malacophyllum* (86 g) were extracted with EtOAc (4 × 150 mL) at room temperature. After removal of the solvent under reduced pressure, 7.5 g of crude extract were obtained and chromatographed over a silica gel column eluted with increasing amounts of EtOAc in hexane. This procedure afforded a fraction (188 mg) purified by prep. TLC over silica gel eluted with hexane/CH_2_Cl_2_ 1:1 to give pure gibbilimbol B (67 mg).

### 3.3. Synthesis of Amides Inspired by Gibbilimbol B

Eight amides (**1**–**8**) chemically inspired by gibbilimbol B were synthesized following a procedure already described in the literature [24]. In general, to a 3-neck round bottom flask, equipped with magnetic stirring at 0 °C and under a nitrogen atmosphere, was added the correspondent amine [octylamine or (*E*)-oct-4-en-1-amine] (1.57 mmol), dry CH_2_Cl_2_ (4.00 mL), and triethylamine (3.14 mmol). Then, a solution of the respective *p*-substituent benzoyl chloride (1.57 mmol) in 4.00 mL of dry CH_2_Cl_2_ was added dropwise to the system, and the mixture was stirred for 16 h. After that, H_2_O (10.0 mL) was added, and the phases were separated. The aqueous phase was extracted with CH_2_Cl_2_ (5 × 10.0 mL). The combined organic phases were then washed with HCl (0.1 mol/L, 3 × 10.0 mL) and a saturated NaHCO_3_ solution (3 × 10.0 mL). The organic phase was dried over anhydrous MgSO_4_, and the solvent was evaporated under reduced pressure, yielding a solid which was recrystallized from hexane to obtain compounds **1**–**8**. All compounds were recrystallized from hexane and fully characterized by ^1^H and ^13^C NMR and infrared spectroscopy, as detailed in the Supplemental Materials (Figures S1–S8).

#### 3.3.1. Methoxy-*N*-Octylbenzamide (**1**)

White solid. IR ν_max_/cm^−1^: 3334, 2954, 2921, 2865, 2852, 1652, 1530, 1500, 1463, 833, 758, 717. ^1^H NMR (CDCl_3_, 300 MHz) δ/ppm: 7.74 (d, *J* = 9.0 Hz, 2H), 6.93 (d, *J* = 9.0 Hz, 2H), 6.08 (br s, 1H), 3.84 (s, 3H), 3.43 (q, *J* = 6.9 Hz, 2H), 1.60 (quint, *J* = 7.2 Hz, 2H), 1.36−1.27 (m, 10H), 0.87 (t, *J* = 7.0 Hz, 3H). ^13^C NMR (CDCl_3_, 75 MHz) δ/ppm: 167.0, 162.0, 128.6, 127.1, 113.7, 55.4, 40.1, 31.8, 29.7, 29.3, 29.2, 27.0, 22.6, 14.1 (Figure S1).

#### 3.3.2. Methyl-*N*-Octylbenzamide (**2**)

White solid. IR ν_max_/cm^−1^: 3336, 2958, 2916, 2869, 2849, 1630, 1528, 1500, 1460, 832, 758, 718. ^1^H NMR (CDCl_3_, 300 MHz) δ/ppm: 7.67 (d, *J* = 8.1 Hz, 2H), 7.23 (d, *J* = 7.7 Hz, 2H), 6.12 (br s, 1H), 3.44 (q, *J* = 6.9 Hz, 2H), 2.39 (s, 3H) 1.60 (quint, *J* = 7.2 Hz, 2H), 1.32−1.27 (m, 10H), 0.87 (t, *J* = 6.8 Hz, 3H). ^13^C NMR (CDCl_3_, 75 MHz) δ/ppm: 167.0, 141.6, 132.0, 129.2, 126,8, 40.0, 31,8 29,7, 29.31, 29.2, 27.0, 22.6, 21.4, 14.1 (Figure S2).

#### 3.3.3. Fluoro-*N*-Octylbenzamide (**3**)

White solid. IR ν_max_/cm^−1^: 3340, 2957, 2917, 2870, 2850, 1631, 1604, 1535, 1502, 1465, 1240, 1159, 849, 768, 727. ^1^H NMR (CDCl_3_, 300 MHz) δ/ppm: 7.78 (d, *J* = 8.7 Hz, 2H), 7.09 (d, *J* = 8.7 Hz, 2H), 6.13 (br s, 1H), 3.42 (q, *J* = 7.1 Hz, 2H), 1.60 (quint, *J* = 7.3 Hz, 2H), 1.33−1.27 (m, 10H), 0.88 (t, *J* = 7.0 Hz, 3H). ^13^C NMR (CDCl_3_, 75 MHz) δ/ppm: 166.4, 162.9, 131.0, 129.1, 129.0, 115.7, 115.4, 40.2, 31.8, 29.6, 29.2, 29.2, 27.0, 22.6, 14.1 (Figure S3).

#### 3.3.4. Chloro-*N*-Octylbenzamide (**4**)

White solid. IR ν_max_/cm^−1^: 3333, 2960, 2919, 2869, 2851, 1632, 1590, 1540, 1472, 1087, 838, 760, 719. ^1^H NMR (CDCl_3_, 300 MHz) δ/ppm: 7.71 (d, *J* = 8.6 Hz, 2H), 7.40 (d, *J* = 8.6 Hz, 2H), 6.20 (br s, 1H), 3.43 (q, *J* = 6.9 Hz, 2H), 1.60 (quint, *J* = 7.3 Hz, 2H), 1.32−1.27 (m, 10H), 0.87 (t, *J* = 7.0 Hz, 3H). ^13^C NMR (CDCl_3_, 75 MHz) δ/ppm: 166.4, 137.5, 133.2, 128.7, 128.3, 40.2, 31.7, 29.6, 29.2, 29.2, 27.0, 22.6, 14.1 (Figure S4).

#### 3.3.5. (*E*)-4-Methoxy-*N*-(oct-4-en-1-yl)benzamide (**5**)

White solid. IR ν_max_/cm^−1^: 3309, 2966, 2930, 2871, 2842, 1631, 1606, 1546, 1502, 1248, 1177, 966, 840, 770. ^1^H NMR (CDCl_3_, 300 MHz) δ/ppm: 7.73 (d, *J* = 8.8 Hz, 2H), 6.92 (d, *J* = 8.8 Hz, 2H), 6.14 (br s, 1H), 5.51−5.36 (m, 2H), 3.48 (s, 3H), 3.45 (q, *J* = 7.0 Hz, 2H), 2.08 (q, *J* = 6.9 Hz, 2H), 1.97 (q, *J* = 6.7 Hz, 2H), 1.67 (quint, *J* = 7.1 Hz, 2H), 1.37 (sext, *J* = 7.4 Hz, 2H), 0.88 (t, *J* = 7.3 Hz, 3H). ^13^C NMR (CDCl_3_, 75 MHz) δ/ppm: 167.0, 162.0, 131.2, 129.2, 128.5, 127.1, 113.7, 55.4, 39.6, 34.6, 30.1, 29.4, 22.6, and 13.6 (Figure S5).

#### 3.3.6. (*E*)-4-Methyl-*N*-(oct-4-en-1-yl)benzamide (**6**)

White solid. IR ν_max_/cm^−1^: 3314, 2963, 2920, 2871, 2854, 1630, 1546, 1510, 1325, 1177, 965, 838, 751. ^1^H NMR (CDCl_3_, 300 MHz) δ/ppm: 7.65 (d, *J* = 8.0 Hz, 2H), 7.27 (d, *J* = 8.0 Hz, 2H), 6.23 (br s, 1H), 5.51−5.36 (m, 2H), 3.45 (q, *J* = 7.0 Hz, 2H), 2.38 (s, 3H), 2.08 (q, *J* = 7.0 Hz, 2H), 1.97 (q, *J* = 6.9 Hz, 2H), 1.67 (quint, *J* = 7.1 Hz, 2H), 1.34 (sext, *J* = 7.4Hz, 2H), 0.88 (t, *J* = 7.3 Hz, 3H). ^13^C NMR (CDCl_3_, 75 MHz) δ/ppm: 167. 4, 141.6, 131.9, 131.3, 129.2, 129.1, 128.8, 39.6, 34.6, 30.1, 29.4, 22.6, 21.4, and 13.6 (Figure S6).

#### 3.3.7. (*E*)-4-Fluoro-*N*-(oct-4-en-1-yl)benzamide (**7**)

White solid. IR ν_max_/cm^−1^: 3296, 2957, 2929, 2870, 1633, 1601, 1547, 1501, 1236, 1177, 970, 850, 760. ^1^H NMR (CDCl_3_, 300 MHz) δ/ppm: 7.78 (d, *J* = 8.8 Hz, 2H), 7.12 (d, *J* = 8.8 Hz, 2H), 6.19 (br s, 1H), 5.52−5.36 (m, 2H), 3.45 (q, *J* = 7.2 Hz, 2H), 2.08 (q, *J* = 7.1 Hz, 2H), 1.97 (q, *J* = 7.0 Hz, 2H), 1.68 (quint, *J* = 7.1 Hz, 2H), 1.35 (sext, *J* = 7.3 Hz, 2H), 0.88 (t, *J* = 7.3 Hz, 3H). ^13^C NMR (CDCl_3_, 75 MHz) δ/ppm: 166.4, 162.9, 131.4, 130.9, 129.1, 129.0, 115.7, 115.4, 39.8, 34.6, 30.1, 29.3, 22.6, and 13.6 (Figure S7).

#### 3.3.8. (*E*)-4-Chloro-*N*-(oct-4-en-1-yl)benzamide (**8**)

White solid. IR, ν_max_/cm^−1^: 3328, 2961, 2929, 2864, 2841, 1633, 1530, 1476, 1248, 1090, 970, 846, 760, 740. ^1^H NMR (CDCl_3_, 300 MHz) δ/ppm: 7.70 (d, *J* = 8.8 Hz, 2H), 7.40 (d, *J* = 8.8 Hz, 2H), 6.14 (br s, 1H), 5.52−5.36 (m, 2H), 3.44 (q, *J* = 7.0 Hz, 2H), 2.08 (q, *J* = 7.1 Hz, 2H), 1.95 (q, *J* = 7.0 Hz, 2H), 1.71−1.65 (m, 2H), 1.35 (sext, *J* = 7.3 Hz, 2H), 0.88 (t, *J* = 7.3 Hz, 3H). ^13^C NMR (CDCl_3_, 75 MHz) δ/ppm: 166.4, 137.5, 133.1, 131.5, 129.1, 128.8, 128.2, 39.8, 34.6, 30.1, 29.3, 22.6, and 13.6 (Figure S8).

### 3.4. Bactericidal Activity of Amides Towards Neisseria gonorrhoeae

The bactericidal activity of the compounds was assessed by determining the minimum bactericidal activity (MBC), through a viable counting method [27]. Briefly, *N. gonorrhoeae* strains were cultured on supplemented GC agar for 16 h, then cultured for 6 h on fresh supplemented GC agar before being suspended in Dulbecco’s Modification of Phosphate Buffered Saline (PBSB, pH 7.4), and transferred to a sterile 96-well plate at a concentration of ~10^3^ colony-forming units (CFUs) per mL. The treatments were dispersed in PBS and added to the bacterial solution at various concentrations. Plates were incubated at 37 °C with 5% (*v*/*v*) CO_2_ for 1 h. Afterwards, 15 µL from each sample were spread onto supplemented GC-agar plates, in triplicate, and incubated overnight to count surviving colonies after 24 h. Bacterial viability was determined by comparing the number of surviving CFUs in treated samples to untreated controls [100 − (cfu/mL with treatment/cfu/mL control) × 100]. Each experiment was performed independently at least three times, and MBC 50% and MBC 90% values were determined from titration curves of % killing against treatment concentration. MBC data in Figure 1 were analyzed using One-Way ANOVA, with *p* values < 0.05 considered significant.

The strains utilized in this study included the laboratory strain *N gonorrhoeae* P9-17 [39], as well as isolates from the CDC/FDA antimicrobial-resistant panel (Strains AR-173, AR-174, AR-187, and AR-200).

## 4. Conclusions

In this study, various amides inspired by the natural product gibbilimbol B were synthesized, and their antibacterial activity was evaluated against *Neisseria gonorrhoeae*. The in vitro bacterial assays revealed that these compounds exhibited notable activity against *N. gonorrhoeae* isolates, including some isolates selected from the CDC/FDA AR antimicrobial panel. This is particularly important given the increasing resistance to ceftriaxone, now the remaining frontline antibiotic for treating uncomplicated gonococcal infections. All synthesized compounds demonstrated superior efficacy in killing *N. gonorrhoeae* compared to the natural product gibbilimbol B. Regarding the structure–activity relationship, the chemical modifications introduced into the gibbilimbol analogs resulted in only subtle differences in antibacterial efficacy. However, the presence of unsaturation at the C-4 position of the alkyl side chain of eight carbons appeared to play an important role in the activity, suggesting that this feature could be further explored in future analog design. Notably, the novelty of this study is that it is the first to examine the anti-gonococcal activity of gibbilimbol B and its analogs, paving the way for further drug modification. Further work to overcome the limitations of our study could include a thorough investigation of the mechanism of action, incorporating computational modeling and wet laboratory testing, such as membrane permeability and protein-binding analysis, to fully understand how these compounds exert their activity. Such studies would also be valuable for the development of new analogs. Additionally, cytotoxicity assays and examination of the potential for gonococci to develop resistance to gibbilimbol analogs would be crucial for evaluating their safety and long-term efficacy as potential therapeutic agents.

## Figures and Tables

**Table 1:** Concentrations of amides **1**–**8** and the natural product gibbilimbol B that kill 50% (MBC50) and 90% (MBC90) of the *N. gonorrhoeae* P9-17 strain. Ceftriaxone was used as a positive control.

Compound	MBC50 (µM)	MBC90 (µM)
**1**	NA	NA
**2**	12.5	25.0 (12.5, 25.0)
**3**	12.5	25.0
**4**	12.5	12.5
**5**	12.5 (6.25, 12.5)	25.0
**6**	12.5 (6.25, 12.5)	25.0
**7**	12.5 (6.25, 25)	25.0
**8**	6.25 (6.25, 12.5)	25.0
Gibbilimbol B	25.0	50.0
Ceftriaxone	2.0	10.0

MBC50 and MBC90: Minimal bactericidal concentrations required to kill 50% and 90% of bacteria, respectively. Values represent the lowest concentration in a 2-fold dilution series that reached the defined bactericidal threshold, based on colony counts. Data are shown as the median of three independent biological replicates (*n* = 3), and the numbers in parentheses are the range of values for the independent experiments; where no parentheses are shown, this denotes that the values were all similar. NA: not active.

**Table 2:** Percentage killing of *N. gonorrhoeae* strains 173, 174, 187, and 200 selected from the CDC/FDA antimicrobial-resistant panel. Gonococcal isolates were treated with compounds **5**, **6**, and **8** at 6.25 µM and 25 µM. Values represent the mean ± standard deviation of three independent experiments, each performed in triplicate.

*N. gonorrhoeae* Strains (AR Bank n^o^)	Compounds					
	5		6		8	
	6.25 µM	25 µM	6.25 µM	25 µM	6.25 µM	25 µM
173	57.0 ± 14.1%	90.5 ± 7.7%	51.3 ± 3.0%	94.1 ± 5.9%	76.4 ± 14.3%	96.9 ± 2.9%
174	44.6 ± 3.0%	85.9 ± 0.5%	44.7 ± 8.2%	93.2 ± 0.1%	65.0 ± 13.9%	88.9 ± 2.7%
187	57.9 ± 9.1%	80.6 ± 6.0%	53.8 ± 1.1%	88.6 ± 5.8%	72.8 ± 3.5%	85.9 ± 3.7%
200	47.3 ± 2.2%	84.7 ± 4.6%	70.2 ± 16.5%	83.9 ± 7.5%	72.2 ± 15.2%	87.0 ± 1.2%

## Data Availability

Dataset available on request from the authors.

## Supplementary Materials

The following supporting information can be downloaded at https://www.mdpi.com/article/10.3390/molecules30112406/s1: Figure S1: ^1^H NMR, ^13^C NMR, and IR of compound **1**; Figure S2: ^1^H NMR, ^13^C NMR, and IR of compound **2**; Figure S3: ^1^H NMR, ^13^C NMR, and IR of compound **3**; Figure S4: ^1^H NMR, ^13^C NMR, and IR of compound **4**; Figure S5: ^1^H NMR, ^13^C NMR, and IR of compound **5**; Figure S6: ^1^H NMR, ^13^C NMR, and IR of compound **6**; Figure S7: ^1^H NMR, ^13^C NMR, and IR of compound **7**; Figure S8: ^1^H NMR, ^13^C NMR, and IR of compound **8**. Figure S9: Titration curves of GIBs against P9-17.
