# Supplementary material for: Bactericidal Effect of Synthetic Phenylalkylamides Inspired by Gibbilimbol B Against Neisseria gonorrhoeae
Source: Molecules. 2025 May 30;30(11):2406. doi: 10.3390/molecules30112406 (PMC12156809; doi:10.3390/molecules30112406)
Supplement: Supplementary file 1 [file molecules-30-02406-s001.zip › molecules-3604796-supplementary.pdf]

Supporting Information

Bactericidal effect of synthetic phenylalkylamides inspired by Gibbilimbol B against *Neisseria gonorrhoeae*

Larissa Verena F. Oliveira[1,2], Andre G. Tempone,[3] Myron Christodoulides[2],

João Henrique G. Lago[1]

[1]*Center of Natural Sciences and Humanities, Federal University of ABC, SP - 09210-580, Santo Andre, Brazil*

[2]*Neisseria Research Laboratory, Molecular Microbiology, School of Clinical and Experimental Sciences, Faculty of Medicine, University of Southampton, Southampton - SO16 6YD, U.K*

[3]*Laboratory of Pathophysiology, Butantan Institute, SP- 05508-040, Sao Paulo, Brazil*

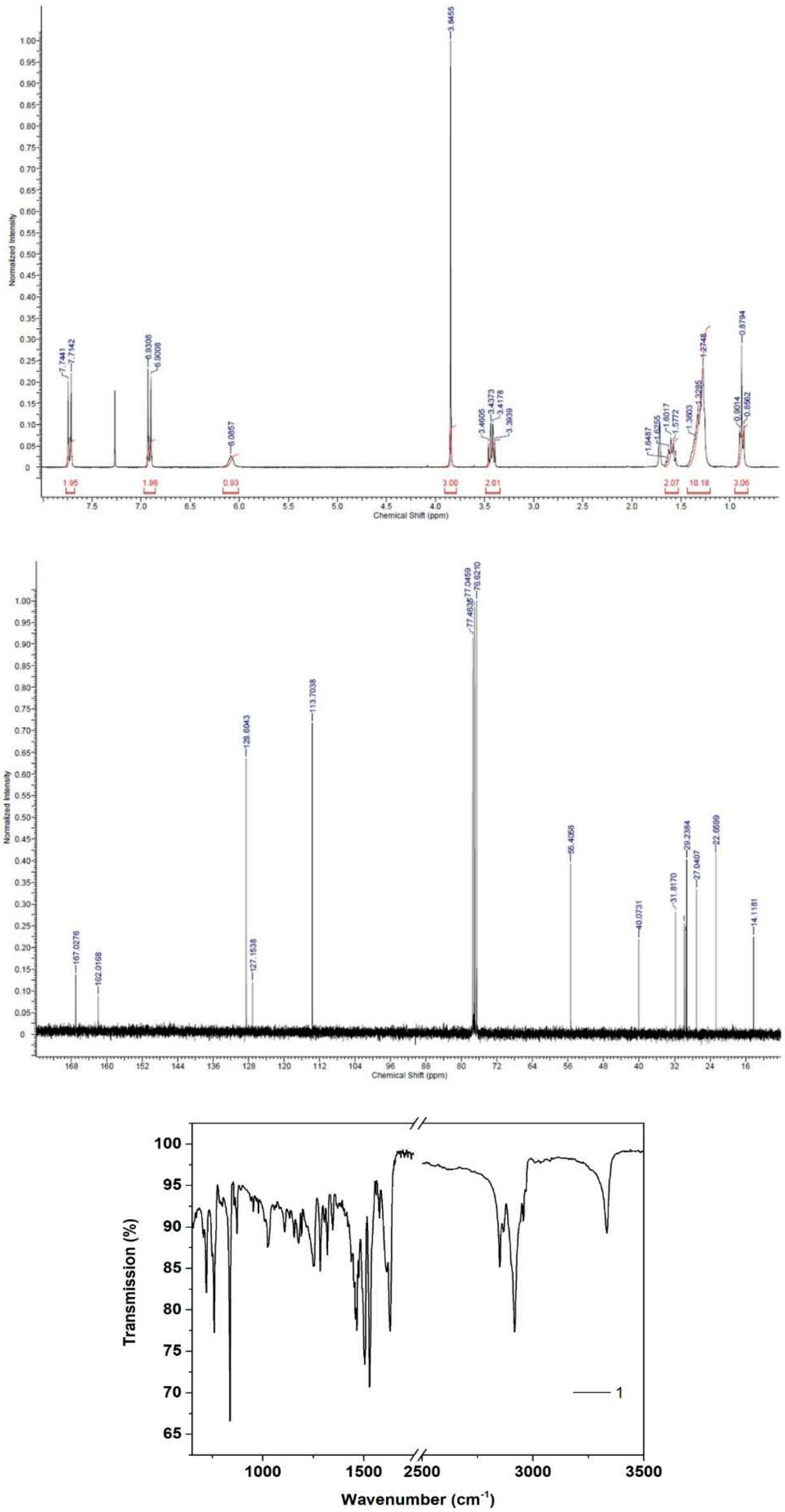

Figure S1: ¹H NMR, ¹³C NMR, and IR spectra of compound **1-** 4-Methoxy-*N*-octylbenzamide

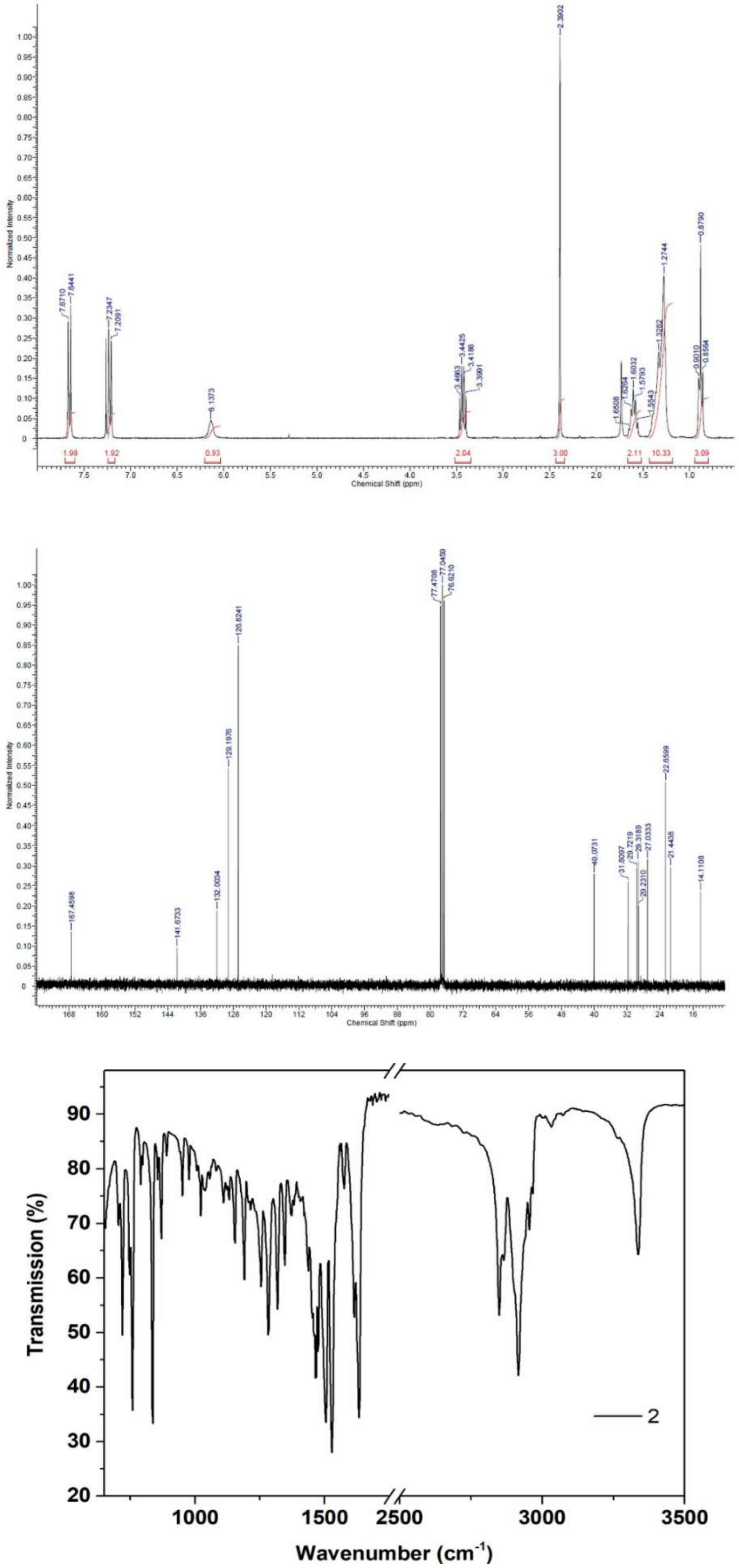

Figure S2: ^{1}H NMR, ^{13}C NMR, and IR spectra of compound **2-** 4-Methyl-*N*-octylbenzamide

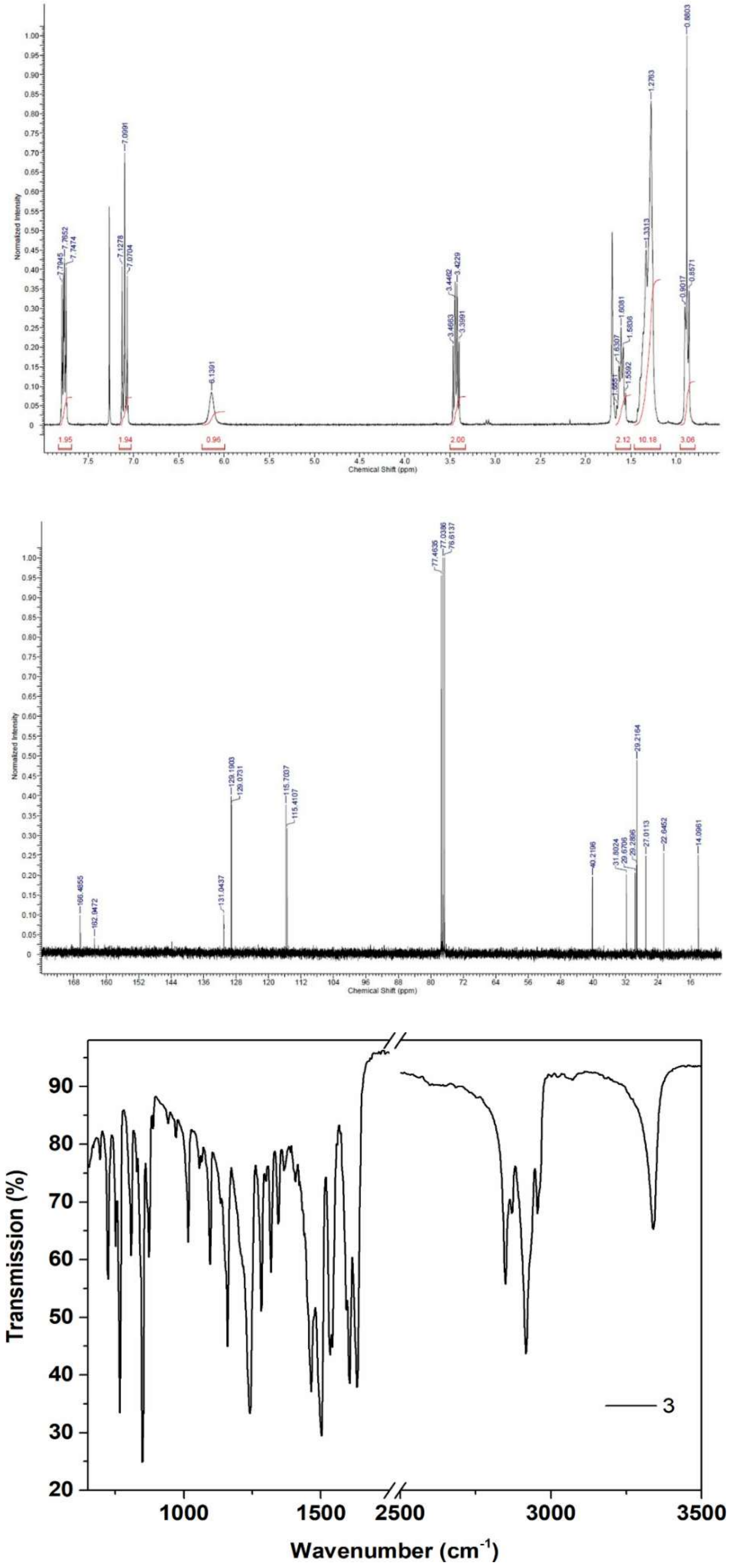

Figure S3: ^{1}H NMR, ^{13}C NMR, and IR spectra of compound **3-** 4-Fluoro-*N*-octylbenzamide

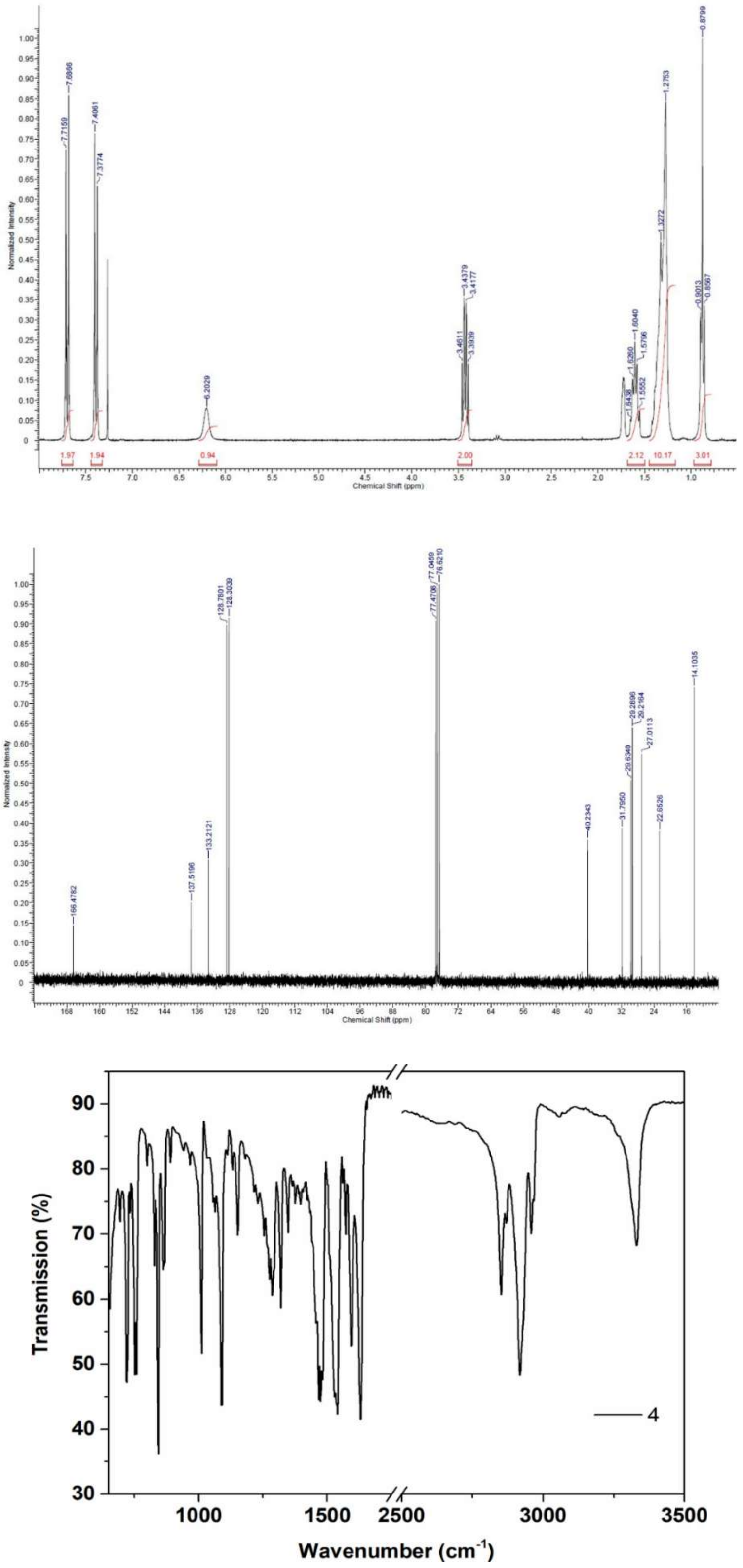

Figure S4: ^{1}H NMR, ^{13}C NMR, and IR spectra of compound **4**- 4-Chloro-*N*-octylbenzamide

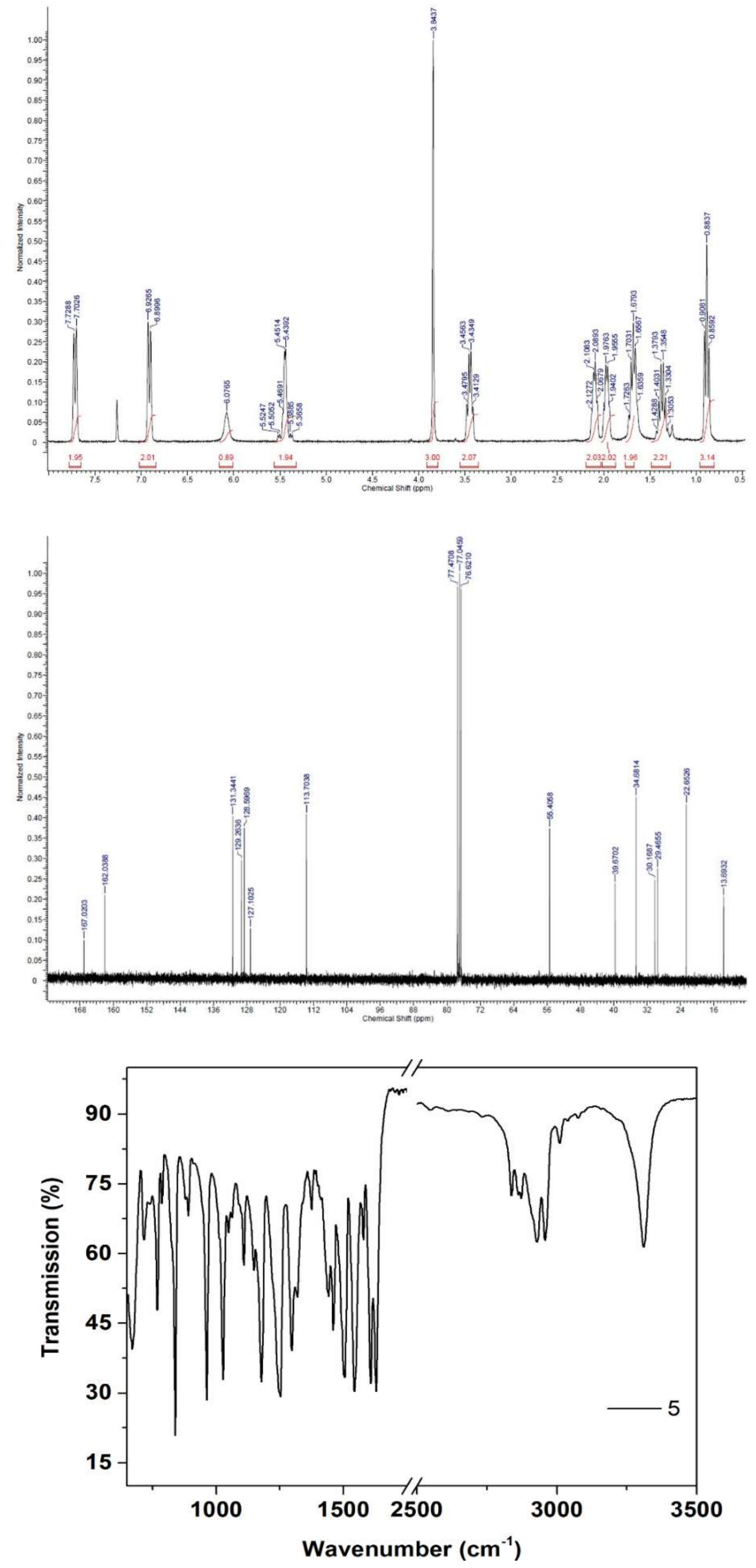

Figure S5: ^{1}H NMR, ^{13}C NMR, and IR spectra of compound **5**- (*E*)-4-Methoxy-*N*-(oct-4-en-1-yl)benzamide

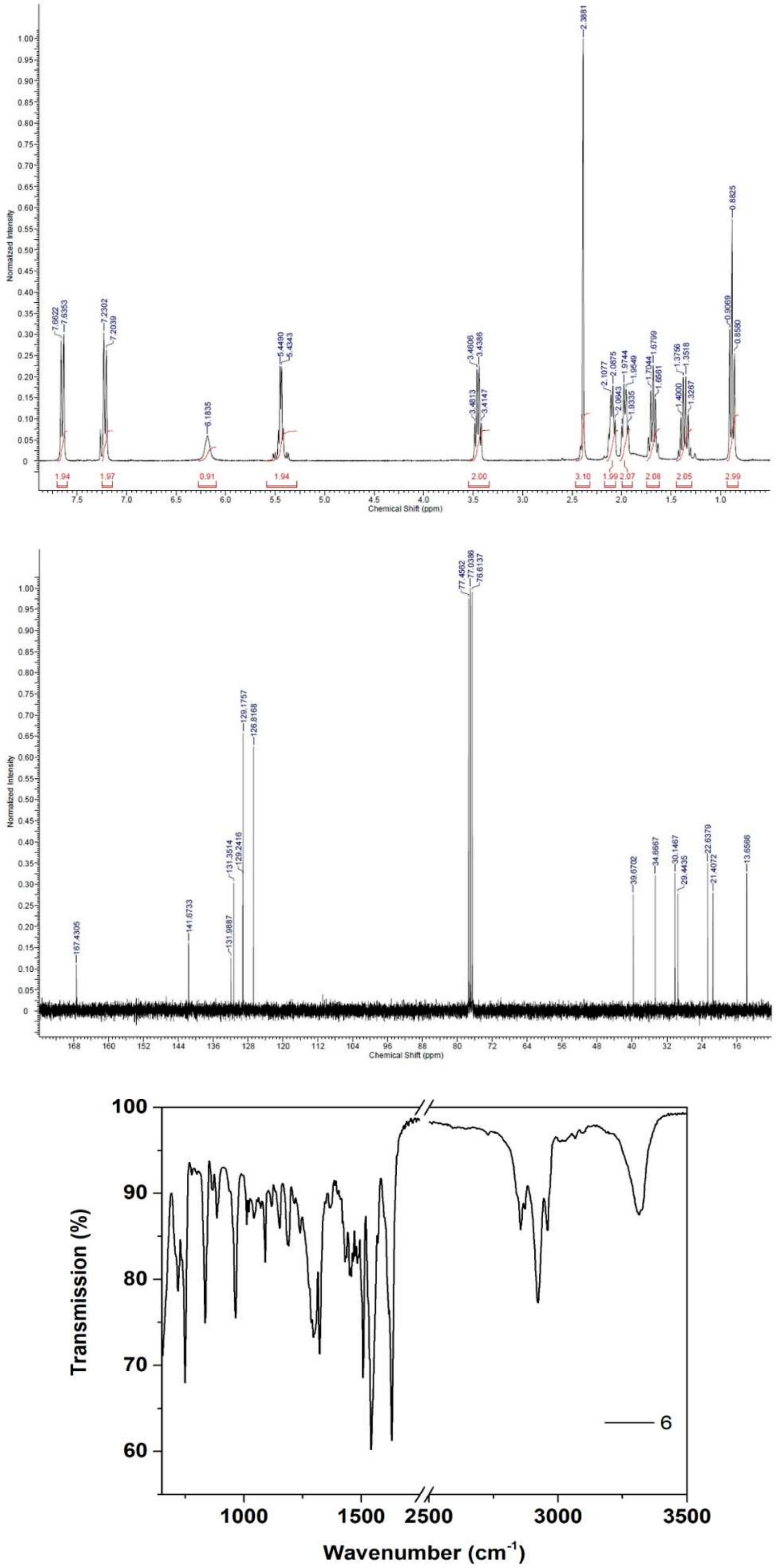

Figure S6: ^{1}H NMR, ^{13}C NMR and IR spectra of compound **6-** (*E*)-4-Methyl-*N*-(oct-4-en-1-yl)benzamide

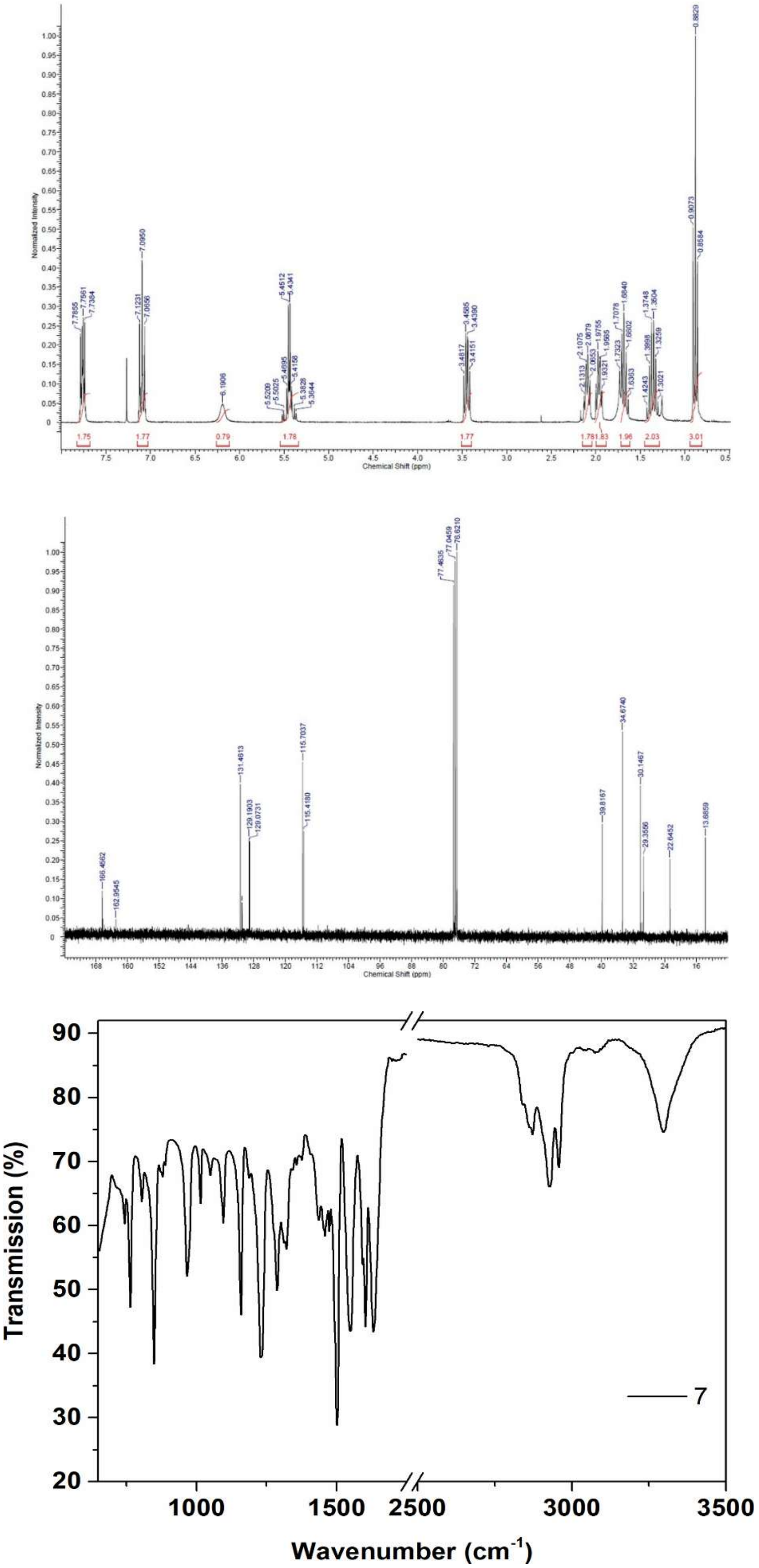

Figure S7: ^{1}H NMR, ^{13}C NMR and IR spectra of compound **7-** (*E*)-4-Fluoro-*N*-(oct-4-en-1-yl)benzamide

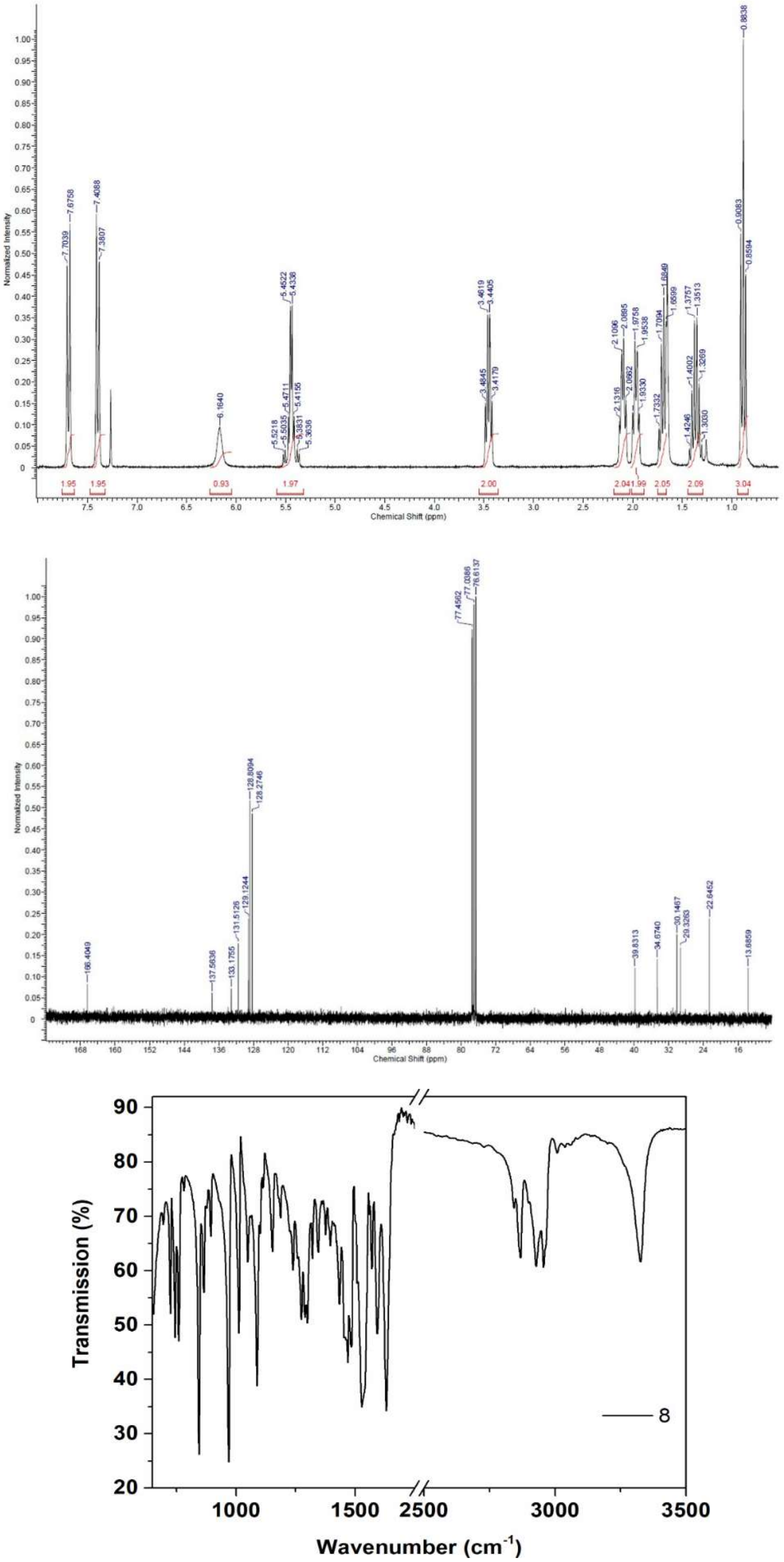

Figure S8: ^{1}H NMR, ^{13}C NMR and IR spectra of compound **8-** (*E*)-4-Chloro-*N*-(oct-4-en-1-yl)benzamide

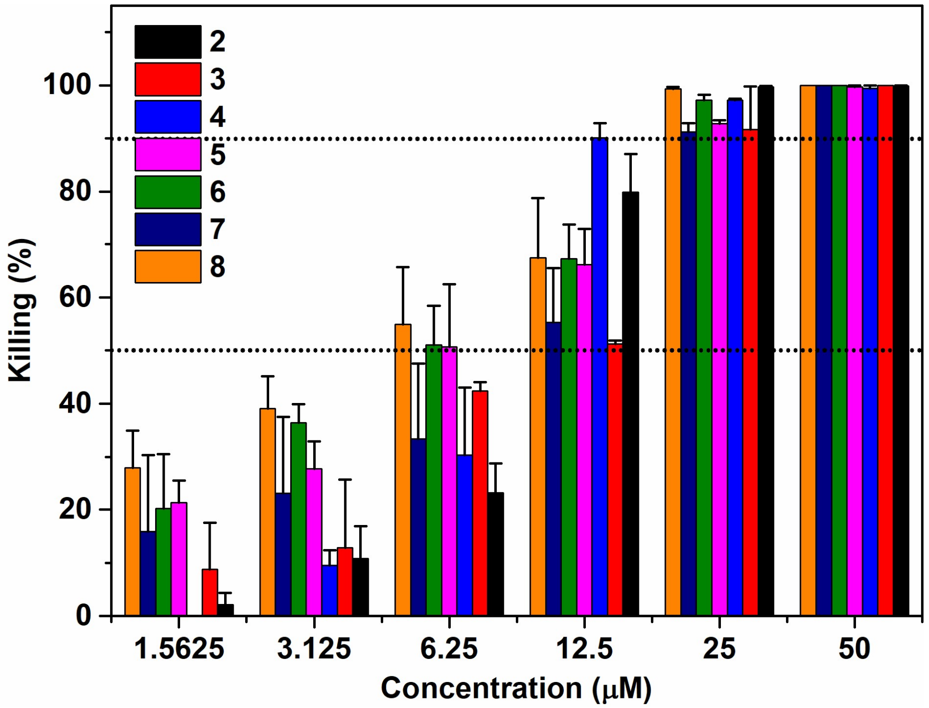

Figure S9: Titration curves: % killing of *N. gonorrhoeae* P9-17 with different concentrations (1.5625 to 50 μM) of the gibbilimbol analogues **2–8**. Each value represents the mean of three independent experiments, performed in triplicate, with error bars indicating the standard error of the mean
